# Protocol for an experimental investigation of the roles of oxytocin and social support in neuroendocrine, cardiovascular, and subjective responses to stress across age and gender

**DOI:** 10.1186/1471-2458-9-481

**Published:** 2009-12-21

**Authors:** Laura D Kubzansky, Wendy B Mendes, Allison Appleton, Jason Block, Gail K Adler

**Affiliations:** 1 Department of Society, Human Development, and Health, Harvard School of Public Health, Boston, MA USA; 2 Department of Psychology, Harvard University, Cambridge, MA USA; 3 Department of Population Medicine, Harvard Medical School/Harvard Pilgrim Health Care Institute, Boston, MA USA; 4 Brigham and Women's Hospital Department of Medicine, Division of Endocrinology, Brigham and Women's Hospital, Boston, MA USA

## Abstract

**Background:**

Substantial empirical evidence has demonstrated that individuals who are socially isolated or have few positive social connections seem to age at a faster rate and have more chronic diseases. Oxytocin is a neurohypophyseal hormone hypothesized to coordinate both the causes and effects of positive social interactions, and may be involved in positive physiological adaptations such as buffering the deleterious effects of stress and promoting resilience. The proposed research will examine whether and how oxytocin influences responses to stress in humans and will consider effects in relation to those of social support.

**Methods/Design:**

Experimental research will be used to determine whether exogenously administered oxytocin (intranasal) influences psychological and physiological outcomes under conditions of stress across gender and age in adulthood. Hypotheses to be tested are: 1) Oxytocin ameliorates the deleterious neuroendocrine, cardiovascular, and subjective effects of stress; 2) Oxytocin and social support have similar and additive stress-buffering effects; 3) Oxytocin effects are stronger in women versus men; and 4) Oxytocin effects are similar across a range of adult ages. Hypotheses will be tested with a placebo-controlled, double-blind study using a sample of healthy men and women recruited from the community. Participants are randomly assigned to receive either oxytocin or placebo. They undergo a social stress manipulation with and without social support (randomly assigned), and outcome measures are obtained at multiple times during the procedure.

**Discussion:**

Understanding the determinants of healthy aging is a major public health priority and identifying effective measures to prevent or delay the onset of chronic diseases is an important goal. Experimental research on oxytocin, social relationships, and health in adulthood will contribute to the scientific knowledge base for maximizing active life and health expectancy. At conclusion of the study we will have solid evidence concerning the effects of oxytocin on stress response and whether it has similar effects across age and gender groups. A neurobiological understanding of resilience can inform efforts for both prevention and intervention of diseases or problems common in later life.

**Trial registration:**

Clinical trial identification number is NCT01011465.

## Background

The biological underpinnings for how positive social relationships may promote health are not well understood. Building on human and animal research that finds early-life nurturing crucial for a range of social, behavioral, physiological, and development outcomes across the lifespan, investigators have hypothesized that social connectedness affects longevity by influencing the rate of aging of the organism. Support for this idea is provided by a large body of epidemiologic evidence linking positive social relationships with greater longevity and delays in functional decline. Oxytocin is a neurohypophyseal hormone hypothesized to coordinate both the causes and effects of positive social interactions. Animal studies have clearly demonstrated a role for oxytocin in the central nervous system whereby it reduces stress-related activation; it inhibits sympathetic nervous system activity and increases parasympathetic activity, and inhibits release of glucocorticoids. There is growing interest in whether the oxytocin system is involved in both social behavior and positive physiologic adaptations related to growth and restorative processes [1-5].

Research on this topic is limited. Much of the work investigating the effects of oxytocin on physiology and behavior in relation to social connectedness as well as stress has been conducted in animals. In addition to their primary findings, these studies have suggested that oxytocin is more potent in the presence of higher estrogen levels, leading investigators to hypothesize that effects of oxytocin are stronger in females than in males. Serious methodological barriers in assessment and measurement of oxytocin in humans present challenges to working in this area. As a result, relevant empirical evidence for physiological and behavioral effects of oxytocin in human populations is still scarce. Of the studies in humans, many have been conducted in samples of either all men or all women with restricted age ranges. Thus, few of these studies have had the opportunity to consider effects of oxytocin in relation to age, or to compare effects of oxytocin on stress and positive social interaction across men and women. This paper describes an experimental protocol in humans that addresses many of these limitations. The protocol is designed to determine whether oxytocin reduces stress-related neuroendocrine and autonomic activation and subjective distress across age and gender, and to examine the effects of oxytocin in relation to those of social support. To make the relevant comparisons possible, the protocol includes men and women across a broad age range. This protocol also breaks through prior methodological barriers that have made it particularly difficult for U.S. investigators to conduct experimental research with oxytocin in humans, by identifying techniques for using intranasal (exogenously administered) oxytocin in a rigorously controlled laboratory setting with human participants.

### Why are Social Relationships Beneficial?

Substantial empirical evidence has demonstrated that individuals who are socially isolated or have few positive social connections seem to age at a faster rate and show evidence of greater physiologic damage over time than those with more positive social connections [6,7]. Much research has focused on negative aspects of relationships, i.e., conflict within or loss of a significant relationship [8-11]. Other stressful aspects of relationships have also been examined. For example, in one study, among mothers of chronically ill children, those who had been caregiving for longer had shorter telomere length, lower telomerase (a cellular enzyme that protects against shortening of telomere-DNA protein complexes) activity, and greater oxidative stress, all markers of accelerated aging [12]. Less work has looked directly at positive aspects of social relationships. Research in this area has broadly suggested that positive feelings generated by close social ties promote health across the life course. For example, in a prospective study of healthy undergraduate men, those who reported feeling more warmth and closeness with their parents during childhood were less likely to suffer from a variety of chronic diseases 35 years later [13]. Moreover, psychological benefits of positive social relationships appear to accrue and persist across the life course [14]. Countering the notion that aging is associated with pervasive loss and sadness, numerous studies have indicated that as people age they tend to increase their involvement in emotionally close relationships (and correspondingly reduce their investment in peripheral relationships). This results in stable levels of positive affect and declining levels of negative affect among older adults [14-17]. Taken together, this work suggests that biological benefits of positive social relationships are likely to be evident even (perhaps especially) at older ages. However, the neurobiological mechanisms by which positive aspects of relationships promote health are somewhat under studied.

### Oxytocin, Positive Social Experiences, and Resilience

Animal research on nurturing and social bonding suggests that oxytocin serves a crucial function in creating a powerful bond between a mother and child. Disruptions to this bond can lead to dysregulation of brain chemistry and specifically the stress response in the baby [18,19]. Recent work also has suggested that dysregulated stress responses early in life are associated with high levels of distress, dysregulated immune response, and other processes in adulthood, which are related to accelerated aging [20]. Oxytocin is a peptide that is produced in a variety of hypothalamic neurons and can be released into the brain or into general circulation in pulsatile fashion by sensory and other stimuli. It is a hormone but also functions as a neurotransmitter. Circulating levels of oxytocin and the number of oxytocinergic neurons in the hypothalamus are independent of gender [21]. Animal research has demonstrated effects of oxytocin not only on a range of social and affiliative behaviors, but also on physiological and developmental outcomes. Oxytocin is clearly involved in general central nervous arousal processes, with effects that reduce stress-related activation [22]. It also seems to shift energy use toward more positive health-promoting internal activities like storing nutrients and increasing the rate of wound healing [23]. Estrogen appears to increase oxytocin receptor gene expression and receptor binding, and animal studies have found that greater oxytocin release occurs in females versus males in response to threat [24-26]. Overall, the research suggests that oxytocin could influence health via two related mechanisms: 1) by promoting social bonding and leading to release of more oxytocin, and 2) by inhibiting stress-related cognitive, affective, and biological activation. The proposed work aims to test aspects of this model concerning potential anti-stress effects of oxytocin and positive social relationships in a controlled laboratory setting.

### Methodological and Conceptual Challenges in Studying Oxytocin in Humans

Human-based studies of oxytocin, social relationships and health are rare, due in part to a complex relationship between central and peripheral levels of oxytocin in humans. Thus, it remains unclear whether mechanisms and effects of oxytocin on social relationships and stress identified in animal models translate to humans and are relevant for human health. Moreover, experimental studies have found that release of oxytocin in the brain is not accompanied by increased oxytocin secretion into the systemic blood circulation, suggesting a separation in the central and peripheral release patterns of oxytocin and presenting additional challenges to conducting epidemiologic research in humans. Some work has examined the interrelationships between oxytocin, social support, and stress in humans; however there are only a few such studies and they are subject to a range of conceptual and methodological limitations. Limitations for this work arise in relation to our ability to obtain valid measures of oxytocin, to assess whether key effects are centrally mediated in the brain versus in the periphery, and the possibility that estrogen or aging alters the potency of oxytocin and its effects

#### Measuring Oxytocin

Findings from research using peripheral measures of oxytocin have been mixed in terms of whether oxytocin is positively associated with better social relationships and less distress [27-35]. However, the variability in the findings may be due to the use of plasma oxytocin measures, which do not necessarily correspond with oxytocin activity in the brain and perhaps should not be interpreted as functioning similarly [33]. Specific biological features of oxytocin limit the applicability of these prior studies. First, effects of oxytocin on general arousal processes are hypothesized to occur via central nervous rather than peripheral mechanisms. Secondly, plasma oxytocin does not easily cross the blood-brain barrier and various factors that influence the release of oxytocin can differ between the peripheral and central oxytocin systems. Experimental studies show limited correlation between oxytocin levels in the blood with concentrations in the brain or the cerebrospinal fluid [36,37]. Moreover, because oxytocin is released into the systemic blood stream in pulsatile form and has a short half-life, it is difficult to accurately assess levels of circulating oxytocin in human plasma without frequent blood sampling. Thus, plasma oxytocin levels may not be a useful indicator of the oxytocin activity in which investigators are most interested. Another way to study oxytocin in humans is to administer it experimentally and directly observe its effects. To circumvent problems with obtaining valid measures of oxytocin, our study experimentally manipulates oxytocin rather than attempting to measure endogenous levels with technology currently available. Experimental manipulations of oxytocin may also provide a better understanding of its effects by providing the opportunity to make stronger causal inferences relative to other study designs.

#### Manipulating Oxytocin

Manipulating oxytocin presents other challenges. When administered peripherally neuropeptides like oxytocin do not readily pass the blood-brain barrier and may also evoke potent hormone-like side effects. However, recent research has suggested that intranasal administration of neuropeptides can bypass the bloodstream and achieve direct access to the brain in both men and women [38]. Also, intranasal effects are more likely to be centrally mediated which mitigates concerns about the relevance of peripheral levels. An intranasal preparation of oxytocin is not commercially available in the U.S. at present which has made it difficult for U.S. investigators to pursue this line of research. Research by other investigators however, suggests that this is a promising line of work [36]. For example, Heinrichs and colleagues examined effects of social support and oxytocin under conditions of stress among healthy young men. Provision of social support and oxytocin each reduced cortisol response and distress in the men, and the combination of both social support and oxytocin had the strongest effects. Another study found intranasal administration of oxytocin increased trust among men [39]. Although baseline endogenous levels of oxytocin were not assessed, findings indicate that studies using intranasal administration of oxytocin may yield insight into the biological processes linking positive social interactions and health.

One concern for studies that look at effects of exogenously administered oxytocin is whether exogenous and endogenous effects are likely to be similar. Prior research suggests that endogenous and exogenous oxytocin have similar peripheral and central effects, including attenuation of behavioral and endocrine responses to stress [40-45]. Taken together, the research suggests that specific effects of oxytocin as an underlying biological mechanism linking socioemotional processes and health can be appropriately investigated using experimental methods with intranasal oxytocin administration [46].

To make it possible to pursue this line of research in the U.S., we have determined that the aqueous form of oxytocin which is commercially available in the U.S. (Oxytocin injection, USP, manufactured by American Pharmaceutical Partners, Inc.) can be used as a nasal spray. The FDA has concurred and they deemed our use of oxytocin injection exempt from requirements for an Investigational New Drug Application (IND).

#### Generalizability of Effects

Much of the experimental research in humans with intranasal oxytocin has been conducted with healthy young men only. Effects of oxytocin may be more difficult to detect in women because oxytocin effects may vary at different points in the menstrual cycle possibly related to estradiol levels. Similarly, only a limited amount of work has considered oxytocin over the life course. One study in humans suggested that younger men may respond to physiologic challenge with increases in plasma oxytocin concentration, but older men do not [47]. Some investigators have speculated that such changes in oxytocin response to challenge may contribute to age-related hypothalamic-pituitary-adrenal (HPA) axis changes such as more prolonged patterns of HPA activation with physiological aging. Empirical evidence however, is not currently available. To gain more insight into effects of oxytocin across gender and age, we are including both men and women ranging in age from 22 to 65 years in our study. We are directly measuring estradiol to consider whether effects of oxytocin vary according to estrogen levels in both men and women, and if such an interaction may account for any observed gender differences in effects.

### Considering Oxytocin, Stress, Social Support, and Health within an Experimental Paradigm

Strong links have been identified between social support and cardiovascular outcomes [48-52] though there is some debate as to whether social support is the critical risk factor versus other psychosocial factors (e.g., depression) that tend to cluster with social support [53,54]. Experimental work has been advocated for understanding observed epidemiologic associations between social relationships and cardiovascular or other health outcomes [55]. Thus building on the epidemiologic findings, numerous laboratory studies have considered the relation between social support and indices of cardiovascular function [50,56,57]. Most of these have focused on the reactivity hypothesis, which posits that exaggerated cardiovascular reactivity (CVR) to stressful experience is one pathophysiologic mechanism linking social stress with the development of cardiovascular disease [58]. While the strongest evidence that CVR is associated with adverse cardiovascular outcomes is from animal studies [50,59,60], there is some suggestive evidence in humans as well [61-63].

Investigators have argued that social support may buffer the cardiotoxic effects of stress by reducing reactivity to acute psychological stress [64]. Generally, these studies have examined whether the cardiovascular and neuroendocrine responses to a stressor depend on type of social support available. This work has consistently found evidence that social support reduces CVR to a stressful experience, although a number of boundary conditions (e.g., friend vs. stranger, passive versus active task, etc) and individual differences (e.g., hostility, defensiveness) have been identified as relevant for whether and when the presence of a supportive person reduces reactivity in a laboratory setting [56,57,65,66]. Consistent with the qualitative reviews of the literature, a meta-analysis of 22 studies suggested that experimental manipulation of social support has medium to large attenuating effects on cardiovascular reactivity measured in the laboratory [56].

### Specific Aims

We are conducting research to gain greater insight into the biological mechanisms underlying epidemiologic evidence linking social relationships, stress, and cardiovascular health. Specifically, our experimental research examines the effects of exogenously administered (intranasal) oxytocin on psychological and physiological outcomes, under conditions of stress. The specific aims of this project are to test the following hypotheses:

1. Oxytocin ameliorates the deleterious neuroendocrine, cardiovascular, and subjective effects of stress.

2. Oxytocin and social support have similar and additive stress-buffering effects.

3. Effects of oxytocin are stronger in women versus men.

4. Effects of oxytocin are similar across a range of younger and older adult ages.

To test these hypotheses, we are conducting a placebo-controlled double blind study using a sample of healthy men and women recruited from the community. We are considering oxytocin effects on a range of outcomes. These include autonomic reactivity as measured by blood pressure responses and high frequency heart rate variability (measure of cardiac vagal tone). Stress-related cardiovascular phenotypes as characterized by the patterning of ventricle contractility, vascular resistance, and cardiac output will also be assessed. Other outcomes include measures of neuroendocrine effects as measured by levels of cortisol and dehydroepiandrosterone (DHEA), subjective distress and positive affect. Participants are randomly assigned to receive either exogenous oxytocin or placebo. They undergo a social stress manipulation with or without social support (randomly assigned), and outcome measures are obtained at multiple times during the experimental procedure. The experiment will test whether effects of oxytocin and social support are similar and additive, and will also compare effects of oxytocin and social support across adult men and women of varying ages.

## Methods/Design

A placebo-controlled, double-blind design is employed. The key experimental manipulation involves the receipt of intranasal oxytocin spray versus placebo (saline) nasal spray. Social support (presence or absence) is also manipulated and age is measured as a continuous factor. Measures of estradiol levels are obtained. Primary outcomes are autonomic and neuroendocrine regulation and affective responses. Effects of oxytocin will be compared with effects of social support, as well as across gender and age. Within-gender group variation in effects of oxytocin will also be considered. This protocol has been approved by the Institutional Review Boards at the two institutions overseeing the research: Harvard School of Public Health and Brigham and Women's Hospital.

### Sample

**Figure 1:**
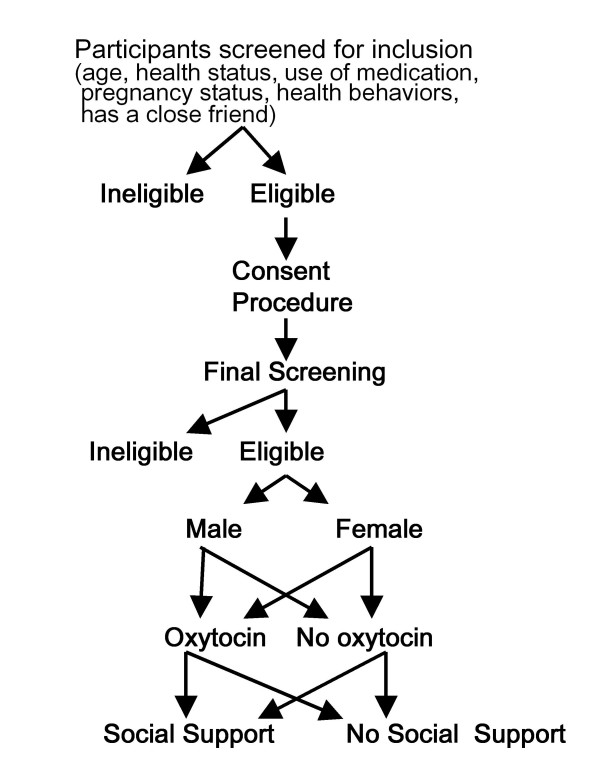
**Sample Recruitment Protocol**.

Participants are recruited from the community via advertising in local newspapers, flyers posted in local areas, and Craig's List. Individuals between the ages of 25 and 65 years are recruited with careful attention to including individuals from across this age spectrum for assignment to oxytocin versus placebo groups. The age range was chosen to capture the spectrum from younger to older adulthood. The older age limit was selected to include those who have entered older adulthood, but among whom there is likely to be a substantial pool of healthy individuals. Interested participants complete an initial telephone screen and a face-to-face screening (see Figure 1).

We conduct extensive screening to determine individuals' health status. Initial screening is conducted on the telephone. For this, participants are informed of the nature of the questions to be asked and the anticipated duration of the screening process (up to 20 minutes). Participants are asked a series of questions about age, medication use, health status, health behaviors, and whether they have a same-sex close friend that could come with them to the laboratory, and will be given basic information about the study. Individuals who have **any **known medical condition (including mental disorders) or are taking **any type **of medication (including birth control pills) are excluded. Because overweight individuals may be at excess risk of undetected diabetes or hypertension, individuals with body mass index (BMI) ≥ 30 kg/m^2 ^are excluded. Further exclusions include pregnancy, breastfeeding, smoking, heavy alcohol use, drug use [as assessed by validated instruments, the CAGE and the RAGS, [67,68], or lack of a close friend who could join the participant for the experiment.

If they are still menstruating, all eligible women are asked to participate during the follicular phase of their menstrual cycle. Study outcomes of interest may be influenced by hormonal fluctuations related to the menstrual cycle. Due to variability in the length of menstrual cycles, it is difficult to determine which phase women are in (luteal, ovulation) and their hormone levels. However, when the menstrual cycle begins, it essentially resets the clock for these fluctuations. Thus we can be confident women are in the follicular phase if we examine them within the first 7 days of starting their cycle. Moreover, at this time hormone levels are relatively low and stable; studies that are concerned about effects of these hormones on other physiological processes are often done during this time [69].

Eligible individuals who agree to participate undergo a brief final screen after arrival at the laboratory and consenting procedures. This is conducted by a physician who obtains a medical history, performs a physical exam, and does a pregnancy test for women. At this time, anyone who is pregnant, has blood pressure values above the normal range (140/90 mm Hg), or meets other medical exclusion criteria is not invited to continue. Eligible participants continue with the protocol. Each participant receives monetary compensation for participation as well as transportation costs. If participant is assigned to bring a friend, the friend also receives monetary compensation for participation as well as transportation costs. Participants are asked to abstain from food and drink (except water) for 2 hours prior to participation in the experiment and from exercise, caffeine, and alcohol during the 12 hours prior to participation. Based on power analyses, the target number of participants per condition is 40.

### Data Collection

**Figure 2:**
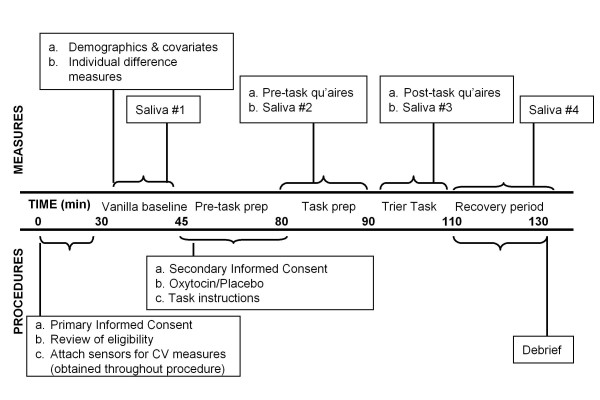
**General Study Protocol**.

This placebo-controlled, double-blind experiment employs a between subjects factorial design, using a 2 (male vs. female) × 2 (oxytocin vs. placebo) × 2 (social support vs. no social support) design. Blinding is utilized for the oxytocin versus placebo groups, but not in relation to who has social support. Participants are naïve as to the experimental hypotheses. Participants are scheduled individually and all experimental sessions are initiated within the same time period (afternoon) to control for diurnal changes in cortisol secretion. Figure 2 shows the timeline for the experimental procedures.

#### Consent Procedures

Primary informed consent is obtained by the research assistant and study physician directly after participants arrive at the Society and Health Psychophysiology Laboratory. This includes consent for the final screening procedures, psychophysiological monitoring, and potential use of oxytocin. If the participant has been instructed to bring a friend, consent for the friend is also obtained at this time. However, consent for public speaking and math performance tasks is delayed to avoid anticipatory stress in the baseline resting period. After the brief final screening, eligible participants are invited to continue. Prior to oxytocin administration written consent is obtained for participation in the public speaking and math performance tasks and for the use of the videotape that will be obtained during task preparation and performance. Participants who refuse to complete the stress tasks are dismissed from further study.

#### Basic protocol

For eligible participants, sensors and transducers are attached for continuous monitoring of autonomic nervous system activity. Participants are asked to complete baseline questionnaire measures assessing relevant demographics and individual differences. After completing these questionnaires participants have a quiet period during which time they read neutral magazines. Following this "vanilla baseline" resting period (found to be effective in reducing effects of extraneous factors), a baseline measure of autonomic indices and the first saliva sample is obtained [70,71]. Consent for the experimental tasks and videotaping is then obtained, and the study physician subsequently administers a single dose of either 24 IU oxytocin or placebo intranasally (approximately 40 min before stress exposure). Following previous work in this area, a dose of 12 IU is sprayed into each nostril [39,72]. In the highly unlikely event of an adverse reaction during the conduct of the experiment, clear procedures have been set up to protect participants.

Task instructions for the stress tasks are given at this time. The stress tasks are initiated 40 minutes after oxytocin or placebo administration, the amount of time other studies have demonstrated it takes for oxytocin to act in the central nervous system [72]. Immediately prior to performing the task, a state mood measure and a second saliva sample are obtained. State mood and post-task measures and additional saliva samples are obtained after completion of the stress tasks. Autonomic function is monitored throughout the procedure. Prior research has strongly indicated that social-evaluative threat is strongest when an evaluative audience is present and the performance is being captured for permanent record [73]. Thus, video cameras mounted in the ceiling record participants' face and upper body during task preparation and performance. In addition to increasing levels of social-evaluative threat, the videotaping procedure also provides an additional method for assessing whether and how participants' responses to social stress varies across conditions; videotapes may be coded later for emotional responses during task conditions by raters blind to study hypotheses or experimental conditions.

At the completion of the protocol, the experimenter assesses whether the subject was suspicious about any aspect of the experiment, explains the nature of the study and debriefs the subject (and friend if present). Participants (and friends, if relevant) are then paid for participation. In summary, the experimental session follows standard procedure for social stress protocols, including a vanilla baseline, task preparation and performance period, and a recovery period. Components of the protocol that are unique to this study are uncomplicated - the administration of oxytocin or placebo prior to task preparation (~1 minute + quiet wait time for it to take effect), the presence of a supportive friend, and the brief screen at the start of the session (~20 minutes in most cases).

#### Oxytocin Manipulation

Building on research suggesting that intranasal administration of neuropeptides bypasses the bloodstream and achieves direct access to the brain [38], we administer intranasal oxytocin, using the biologically active form of the neuropeptide. Intranasal oxytocin has been widely prescribed in pregnant women to help induce labor and delivery, and in non-pregnant women to promote lactation. In non-pregnant women, intranasal oxytocin is typically self-administered by patients multiple times over the course of the day for several weeks at a time in a non-medical setting (e.g., in the patient's home prior to breast feeding) and is well tolerated. Experimental studies of effects of oxytocin have been conducted in men as well as women, with intranasal doses between 20 and 264 IU, and no adverse side effects have been reported [74-79]. Moreover, studies in humans on memory, affect, and biological parameters have clearly demonstrated behavioral and psychoneuroendocrine effects using a dose of 24 IU intranasal oxytocin (the dose we are using) [39,72,80-82]. No adverse or unanticipated side effects have been reported in these studies. While studies of intranasal oxytocin have largely focused on younger adults (ages 18 to 25), a number of studies have included older adults with and without mental health problems and reported no concerns around the use of intranasal oxytocin [79,83].

In this study, participants are randomly assigned to receive either oxytocin or placebo intranasally with both investigators and participants blind to condition. Participants assigned to the oxytocin condition receive a dose of 24 IU oxytocin and participants in the placebo condition receive saline. The Brigham and Women's Hospital Investigational Drug Pharmacy is responsible for obtaining the oxytocin and placebo and preparing them for administration to participants. For participants receiving oxytocin, aqueous oxytocin is inserted into a spray bottle. The spray bottle is calibrated so that emptying the spray bottle results in delivering 24 IU of oxytocin, with about half the bottle sprayed into each nostril. No dilution of the original aqueous form of the oxytocin is necessary. The nasal spray (oxytocin or placebo) is prepared within 24 hours of administration. Duration of drug administration is approximately 2-5 minutes.

#### Social Support Manipulation

Support manipulations have varied across studies, with some studies requiring support providers to be in physical contact with participants [84,85] vs. activating support schemas [86], others asking support providers to give verbal support [66,87], use of confederates vs. friends to increase standardization of support provided [87-89] and various strategies employed to reduce potential for evaluation [85,90]. Findings generally suggest that reduced potential for evaluation, having a genuine relationship with the support provider, and verbal support are key components [56,57]. Building on prior work and our own pilot research, the social support condition is designed to standardize the support available to participants across individuals, as well as to maximize the effectiveness of the support provided. Prior to coming to the laboratory, participants are randomly assigned to one of two conditions, requiring them to appear alone or accompanied by a friend. They are then instructed to either bring a same-sex close friend (non-romantic, excluding spouses) with them, or to come alone. Same-sex friends are recruited to reduce variability associated with the meaning of their interactions. Upon arrival at the laboratory, individuals and their friends/support providers are introduced to the study and consented together. Friends then stay in a waiting room, and join participants at the start of the stress exposure when task instructions are given. All participants in the friend-present condition are told that the friend's role is to be "a support partner," and "to silently cheer him/her on." Friends sit where they can see the participant but not directly facing him or her. To control interaction between the participant and friend all remarks by the friend are scripted. Friends are asked to make an encouraging remark when they join participants and when participants finish each task. To minimize possible evaluation effects, friends are given their own tasks to complete (fill out questionnaires) and asked to wear a headset playing white noise during task performance. Friends are instructed not to distract participants at this time, but are given a stop watch and asked to nod and smile briefly at the subject at 1-minute intervals during each task. Friends leave participants at the start of the recovery.

#### Social Stress Manipulation

Social stress is induced using the Trier Social Stress Test (TSST), which prior work has suggested effectively creates a stressful situation that might be encountered in the real world [91]. The TSST generally requires participants to perform two separate tasks in front of an audience: a 5-minute public speaking task and a mental arithmetic exercise. Modifications have been made to ensure the effectiveness of the manipulation for all ages. For the speech task, participants are asked to prepare a speech about important self-relevant topics. All participants (regardless of age) are given the same 4 topics (Social Security reform, cost of prescription drugs, education reform, or the cost of gasoline) and asked to be prepared to present their thoughts about all the topics and potentially to answer questions about the topics from the evaluator. They are asked to try to talk for the full 5 minutes with as few pauses as possible. They are informed that the talk will be videotaped for later evaluation and that evaluators will be coding verbal and non-verbal behavior to assess their ability to successfully present their thoughts. They are further told that the evaluators have had extensive training on speech evaluation, are very skilled in assessing non-verbal behavior and body language, as well the persuasiveness and coherence of the speech. After the speech task, participants are asked to perform an arithmetic task, which is described as being related to mental ability and general intelligence. Participants are instructed to count backward from 996 by 7. Each time they make a mistake the evaluator informs them they have made an error and must start again. Participants are informed that they will be evaluated based on how many errors they make, how quickly they perform the task, and their overall presentation. As social support has been shown to be more beneficial when social threat is high [56], evaluators are trained to act in an impersonal fashion and to remind participants that they are being evaluated, to increase the social threat inherent in the task.

#### Biological Stress Responses

Multiple regulatory systems interact via a non-linear network to enable individuals to adapt to challenges and stressors in their environment. Exposure to acute stress elicits a cascade of cognitive, affective, and biological responses. Moreover, according to the biopsychosocial model cardiovascular responses may be either adaptive or maladaptive [92,93]. When situations are construed as threatening, cardiovascular responses show more vascular increases, as evidenced by increased ventricular contractility, heart rate, and total peripheral resistance with little change in cardiac output. When situations are construed more positively, cardiovascular patterns are also marked by increased ventricular contractility, heart rate, and cardiac output, but with decreases in total peripheral resistance. Cardiovascular responses consistent with more positive versus threatening appraisals have been linked with better outcomes -- lower anxiety, improved hormone functioning and higher telomerase activity -- and are considered more benign [94-96].

In a healthy or resilient system, a variety of regulatory mechanisms are maintained to counteract or speed recovery from stress-related activation. In a less resilient system, repeated and frequent activation of processes that are adaptive in the short-term may lead to cumulative systemic damage and ultimately initiate disease-related processes [97]. Vagal tone is hypothesized to be critically important in psychological and physical health. Porges (2003) argues that greater vagal control is associated with emotion regulatory capacity and possibly more positive social emotions and awareness of the social environment. Similarly, lower vagal tone has been related to depression, hostility, and anxiety [98-101]. Greater vagal control has also been associated with reduced risk of heart disease, relapse after myocardial infarctions, and reduced cellular aging [102-104]. If oxytocin and social support either directly increase vagal tone and physiologic resilience, or buffer the effects of stress, we might expect to see significantly more benign cardiovascular reactivity and higher levels of heart rate variability in response to a stressor and/or increased basal levels of parasympathetic tone. Each of these cardiovascular responses is measured over the course of the experiment.

Another index of regulatory capacity considers the neuroendocrine system. Previous work has found that the hormone cortisol (released by HPA axis activation) is highly reactive to stress, and chronically high levels of cortisol have been linked with risk for a variety of poor health outcomes including cardiovascular diseases and decreased immune functioning (although hypocortisolism has also been identified as problematic). Less studied but of growing interest, is the class of anabolic hormones which counter-regulate catabolic hormones and provide indications of physical and psychological thriving and greater adaptive coping [105]. One such anabolic hormone is DHEA and its active metabolite DHEA sulfate (DHEA-S), which is excreted by the zona reticularis of the adrenal cortex in response to adrenocorticotropic hormone (ACTH, part of the HPA axis cascade that directly precedes cortisol production). Epidemiological data has revealed relationships between low levels of DHEA and DHEA-S and cancer, age-related disorders, immune functioning, and also mental health outcomes, especially depression [106]. The ratio of anabolic to catabolic hormones provides additional information regarding the net anabolic vs. catabolic effect on the body, and is a more sensitive indicator of well-being than either measure alone. For example, androgens like DHEA can counter catabolic effects of cortisol on immune function, neurons, and on protein synthesis. The net ratio provides a measure called 'anabolic balance' (operationalized by DHEA/cortisol) [103,105,107]. This has been related to better performance, fewer depressive symptoms, and greater positive well-being [95,108]. We collect saliva at several points during the course of the experiment so that we can assess the effect of the experiment on hormonal response.

### Measures

#### Autonomic Nervous System (ANS) Activation

Measures of ANS activation include parameters that are either directly measured or that can be derived from primary signals. Thus, measures of systolic and diastolic blood pressure and mean arterial pressure are obtained as well as heart rate, cardiac output, total peripheral resistance, pre-ejection period, and heart rate variability. Cardiac and hemodynamic measures are recorded noninvasively according to established guidelines for impedance cardiography and blood pressure measurement [109]. Condition averages of the cardiac and hemodynamic measures are derived from the electrocardiogram (ECG) and blood pressure monitor. For each measure, scores from the baseline condition will be compared with task condition scores to indicate reactivity [110]. Vagal control is assessed via respiratory sinus arrhythmia (RSA) which will be derived from both ECG recordings and respiration estimated from impedance waveforms (ZKG). A computer program (Mindware) is used to identify R spikes and R-R intervals in the ECG waveform with inaccurately identified R spikes manually corrected. The data are detrended and submitted to Fast Fourier Transformation, and the high-frequency power spectrum (0.15-0.40Hz) is used as an index of RSA [111]. Because we are studying a healthy population and reactivity is not a clinical endpoint, we will consider whether patterned responses during and after the task are consistent with a maladaptive vs. an adaptive response. Following Porges' work we will interpret greater vagal control as a more adaptive regulatory response. While statistical and clinical significance may not be the same, statistically significant effects in these experiments would provide important evidence of an underlying biological mechanism for the beneficial effects of social interactions and the role of oxytocin in this process.

#### Hormone Measures

Salivary hormone concentrations reflect the unbound serum hormone fraction and are considered a reliable and valid measure of the free fraction concentration of the hormone in the blood [112,113]. Saliva samples are collected using IBL sampling devices and the methodology described by Kirschbaum and Hellhammer [114]. Four saliva samples are collected from each participant over the course of the experimental session. After participants expectorate 1 ml of saliva into the Sali-caps, each saliva sample is stored (in -80 degrees C) until required for biochemical analysis, when they are sent to a laboratory to be assayed. A random sample of 20% of the samples will be assayed in duplicate. Anabolic balance will be derived from assay results by creating a ratio of DHEA/cortisol after molar transformation to equate unit values [107]. Estrogen is measured using estradiol obtained from the first saliva sample taken at the laboratory session [113,115].

#### Psychological Measures

Negative emotions are assessed with the Spielberger State-Trait Personality Inventory (STPI), assessing symptoms of anxiety, anger, and depression [116]. Trait measures assess stable individual differences in the frequency and intensity with which individuals experience these negative emotions. Subscales all have high a coefficients (all < .75) and demonstrated validity. State measures assess how anxious, angry or depressed individuals feel while in the laboratory, and will be given just prior to performing the speech task and again after completion of the math task. State and trait positive affect are measured using the Positive and Negative Affect Schedule (positive affect subscale) which prior studies have indicated is both valid and reliable [117]. Because some studies have indicated that hostility, defensiveness, or attachment style may affect the relationship between social support and CVR, we include measures of these constructs [66,118,119]. Hostility is measured with the Cook-Medley hostility scale, which has demonstrated reliability and validity, and has been found to predict coronary heart disease [120,121]. Defensiveness is measured using the Marlowe-Crown Social Desirability scale, following other work in this area [66,122]. Attachment is measured using the Experiences in Close Relationships measure [123].

Because basal levels of oxytocin may be influenced by general availability of social support, prior to the experimental tasks we also evaluate perceived social support with the Interpersonal Support Evaluation List, which measures perceived availability of social support resources [124]. This permits assessment of the adequacy of existing social support networks, which could conceivably affect people's response to the manipulations. If a support provider is present, prior to the experimental tasks we also ask participants about the length and nature of their relationship with the friend. After participants complete the experimental tasks we ask about their perceptions of the extent to which the friend was supportive during the stress tasks [89]. All participants are also asked to report how involved they felt, and how much difficulty they experienced while performing the tasks.

### Equipment

The psychophysiology laboratory at HSPH includes equipment for assessing cardiovascular function within a four-room suite that includes a control room and rooms for collecting physiological and other types of data. It is equipped with a Biopac MP150 system (BIOPAC Systems Inc, Goleta, CA) for the measurement of ECG, impedance cardiography, and blood pressure. It is further equipped with a stadiometer, stimulus presentation equipment, and a complete audio-visual system for monitoring and data collection (including multiple video cameras, speakers, etc). The laboratory also has capacity for extraction and storage of biological samples (e.g., blood, urine, saliva). Blood pressure is recorded with a Medwave blood pressure monitor that provides continual readings of blood pressure. Cardiac responses are obtained with ECG system using a Standard lead II configuration (ECG100C) and an impedance cardiograph system (NICO100C). The impedance cardiograph uses a four mylar band configuration placed around the neck and torso and was developed according to published guidelines [125]. All signals are conditioned, amplified, and recorded onto a computer for subsequent editing and ensembling. The editing program (Mindware Suite, Mindware Technologies, Westerville, OH), is a specialized scoring program that allows for artifact editing and ensembling averages of impedance waveforms.

### Data Analyses

Analysis will begin with thorough descriptive and graphical examinations of variables in both studies, including comparisons across randomization groups. Analyses will include adjustment for covariates if not covered by randomization. Distributional assumptions of dependent variables will be assessed, and measures will be transformed, if necessary. Analyses will use applications of the general linear model as appropriate for each hypothesis under study. For example, when examining relations between the dichotomous factors of social support, oxytocin, and gender, we will use analyses of variance (ANOVA) with repeated measures. Responses are measured over time both before and after the stress induction. Cardiovascular outcomes will be considered both in separate models and using multivariate analysis of variance, as they are likely correlated within each individual. When considering the relation between age (a continuous factor) and oxytocin, we will use multiple linear regression techniques incorporating an interaction term between age and oxytocin as well as relevant covariates, but we will also consider using linear mixed models to account for the repeated measures on the outcomes and the resulting intra-individual correlation in these measures. Statistical tests will be two-tailed and conducted at the .05 level of significance. Regarding the specific hypotheses, this experimental study provides data for analyses that will examine whether:

1) Under conditions of stress, higher levels of oxytocin lead to higher vagal control, more benign stress reactivity (reduced blood pressures, cardiovascular response patterns characterized by increased ventricular contractility, heart rate, and cardiac output, but decreased total peripheral resistance), higher anabolic balance, and reduced subjective distress, by comparing the change in responses before and after stress across the oxytocin and placebo control groups.

2) Oxytocin combined with social support reduces the effects of stress more than the individual effects of oxytocin and social support by comparing the change in responses before and after stress across all possible combinations of the two treatment groups (oxytocin vs. placebo; social support vs. no social support). Oxytocin and social support combined is expected to be associated with highest levels of vagal control, the most benign pattern of autonomic reactivity, and the lowest levels of subjective distress.

3) Oxytocin reduces effects of stress more for women than for men by comparing changes in responses before and after stress in the presence of oxytocin or placebo across men and women. Exploratory analyses will consider whether the strength of the effects of oxytocin may be attributable to differences in estrogen levels by comparing effects of oxytocin across pre- and post-menopausal women (estrogen levels are expected to be higher among pre-menopausal women) and also considering interactions between estrogen (measured directly via levels of estradiol) and oxytocin in both men and women.

4) Effects of oxytocin are similar across younger and older aged adults by comparing the change in responses before and after stress in the presence of oxytocin or placebo across age.

The general analytic strategy will start by fitting a model that includes all interactions and then move to a statistically equivalent simpler model using likelihood ratio tests. In cases where there are more than two measurements over time, several covariance structures will be considered. The possibility of multiplicative effects (between oxytocin and social support, for instance) will be explored graphically. Other research has suggested that the effects of oxytocin and/or social support may not emerge immediately. Thus, elapsed time will be an important consideration, and separate analyses will be conducted to determine when over the course of the experimental tasks, effects of oxytocin and/or social support are evident on cardiovascular, neuroendocrine, and subjective parameters. These analyses will involve multiple single comparisons between baseline and later levels of the dependent variables, and will be conducted with adjusted levels of significance as appropriate (e.g., t-tests with Bonferroni corrections).

Additional analyses will use separate models testing interaction terms to consider whether stable characteristics of the individual (i.e., hostility or defensiveness, general levels of perceived social support, nature of relationship with the support provider) may alter effects of either oxytocin or social support on responses to stress. Population based studies that have looked at the distribution of hostility or defensiveness suggest that there is a broad range of scores that are skewed toward lower levels, even in populations with somewhat higher mean scores [126,127]. Moreover, individuals with high levels of these traits tend to have lower levels of social support [66,126], making it less likely that they would have a close friend and would therefore be excluded from the study initially. As we are randomizing individuals to support or no support, and cell sizes are moderate (n = 40), it is not unreasonable to expect a relatively equal distribution across the conditions of individuals with specific characteristics that might affect responses to the social support manipulation. We will however, control for these individual differences in the primary analyses. Additional analyses will consider effects of individual differences by stratifying across individuals who are high and low in a particular trait and examine for trends that might suggest possible interactions. We will also examine if perceptions of support provided during the experiment are influenced by oxytocin. As the support provided is highly standardized, perceptions of greater support may suggest that oxytocin enhances receptivity to support.

### Power and Sample Size

Power is calculated to accommodate both the effects of the dichotomous experimental factors, as well as age (measured as a continuous variable). Since power will be limited by hypotheses where 3-way interactions are being tested, power is first computed for representative outcomes for those hypotheses (including vagal tone measured by RSA), based on information available in the existing literature. Power for hypothesis 1 will be higher. Power is calculated for a two-sided test of a three-way interaction over ranges of reliabilities and differences to be detected. For all outcomes, 40 participants per gender/treatment group will result in power of 80-90% to detect effect sizes similar to those seen in the literature. Since participants are used as their own controls, power varies by measurement reliability, and this sensitivity was considered. Power was computed using a normal test that assumes independence across participants, compound symmetric covariance within each subject, and approximately constant variance before and after the treatment. Values for the parameters used here were obtained from the literature [72,88,89,98,128]. In addition, a sample of this size will provide 80% power to detect a medium sized effect using multiple linear regression to consider age and oxytocin adjusting for other covariates as necessary [129].

## Discussion

Understanding the determinants of healthy aging is a major public health priority and identifying effective measures to prevent or delay the onset of chronic diseases is an important goal. Limited work in human populations has focused on protective factors that promote resilience in the face of challenge over the life course, or has included consideration of these processes at the molecular level. Taken together with recent theories of biobehavioral mechanisms underlying both stress and health benefits of positive social relationships [2,5,130], molecular work implicates oxytocin and endogenous opioids in regulating stress-responsive systems or stimulating other internal regulatory systems involved in social interactions and health. However, empirical evidence for these ideas in human populations is still exceedingly limited, and insight at the molecular level has largely been provided by brain research in animals, with active investigation of the effects of oxytocin on physiological and behavioral stress responses.

Experimental research on oxytocin, social relationships, and health in adulthood will contribute to the scientific knowledge base for maximizing active life and health expectancy. The proposed research on oxytocin and social relationships integrates concepts of life-course development, and represents a critical domain that may contribute importantly to improving health and well-being at older ages. This work represents the earliest stages of investigation, but begins to address questions about oxytocin and how it may help to explain the links between social relationships and aging. This study will be one of the first to include older individuals in the study and consider combined effects of oxytocin and social support on stress response in relation to age. Moreover, despite hypothesized differences in effects of oxytocin related to estrogen, this will be one of the first studies to compare the effects of oxytocin on stress and positive social interaction across men and women. Most experimental studies have been conducted in samples of either all men or all women. Additional insight will be gained by considering effects across multiple domains, including a detailed consideration of cardiovascular stress response with a particular focus on parameters that have previously been linked with risk of developing heart disease. Identification of how a specific biological substrate is involved in the relationship between positive social and emotional factors and physical health in older men and women will provide a more definitive understanding of healthy aging processes.

### Potential Limitations

There are several potential limitations to this study. First, the animal literature suggests that effects of interest seem to be a function of brain levels of oxytocin rather than levels in the periphery. However, oxytocin released in the brain cannot easily be measured, and measures of oxytocin in blood plasma that can be obtained may not be functionally related to the key biological effects of interest. Relatedly, with intravenous administration neuropeptides cross the blood/brain barrier, but at such low rates that a pharmacological dose would be needed to achieve behavioral effects [131]. Such high doses would create confounding peripheral influences that would make mechanistic interpretations difficult. Making use of recent work indicating that intranasal administration of neuropeptides can gain access to the brain and produce central effects [38,132], we have broken the barrier for conducting this work in the U.S. by establishing that the aqueous form of oxytocin (commercially available in the U.S.) can be used with a nebulizer as a nasal spray.

Second, we cannot directly measure levels of oxytocin either at baseline or after intranasal oxytocin administration. Thus, we cannot fully account for basal oxytocin levels nor confirm levels of oxytocin in the brain after administering the hormone. Due to the invasive nature of the procedure, few experimental human studies of oxytocin have measured cerebrospinal fluid (CSF) for oxytocin levels either prior or subsequent to intranasal administration of the peptide. However, studies that have administered oxytocin intranasally have reliably found the predicted effects despite the inability to confirm levels of oxytocin in the brain after administering the hormone and regardless of basal oxytocin levels [39,72,133]. A salivary assay for oxytocin is under development [134] but is not yet available; we hope to be able to incorporate use of this biomarker with subsequent studies in this line of work. However, based on the existing literature, we anticipate that our current methods will provide valid evidence on which to build future studies.

Third, we cannot determine whether exogenous administration of oxytocin is equivalent to endogenous oxytocin. Human studies using exogenous intranasal administration of oxytocin have produced findings highly consistent with those in the animal literature examining effects of both exogenous and endogenous oxytocin [46]. We are administering the biologically active form of the neuropeptide. Endogenous and exogenous oxytocin have similar effects on outcomes including with regard to potential anxiolytic effects, attenuation of behavioral and endocrine responses to stress, and social and reproductive competency [41-45]. For example, endogenous central and peripheral oxytocin are elevated during lactation, and physiological responses to stressors are attenuated [42,43,135-137]. In rats, exogenous oxytocin reduces corticosterone secretion in response to stress and decreases anxiety [45,138]. There is also evidence in rats that early exposure to either exogenous or endogenous oxytocin has long-lasting organizational effects in relation to expression of adult behavior [139-141]. Studies of effects of endogenous oxytocin during lactation in humans have been used [27,135], but it is difficult to control confounding factors in endogenous stimulation paradigms, particularly the release of other hormones [72]. Moreover, the inhibitory effect of oxytocin on HPA axis responsiveness appears to be a function of central modulation. Taken together, the research suggests that specific effects of oxytocin as an underlying biological mechanism linking socioemotional processes and health may be appropriately investigated using experimental methods with intranasal oxytocin administration [46].

Fourth, generalizability is limited because we include only healthy community volunteers. Our sample includes individuals of all races and ethnicities who are eligible. Ideally this will result in a racial/ethnic distribution in the sample that approximately mirrors the locales from which we will be recruiting. However we recognize that in fact, it may be more difficult to recruit minority participants which could result in a sample that is not representative of the local community. Future work will be needed to determine whether effects are similar across diverse populations and in the more general population.

Fifth, we will not be able to determine how oxytocin specifically influences cognitive and affective processes related to fear and anxiety or if effects of intranasal oxytocin are mediated by central or peripheral increases. It may be possible to gain greater comprehension of the mechanisms underlying these processes as new techniques for obtaining peripheral measures of oxytocin are developed. Additional work may also develop animal models to help address these questions, with the animal studies explicitly building on the finding from and gaps noted in the human studies.

### Future Directions

This research uses a multidisciplinary approach to examine neurobiological underpinnings for the observed epidemiological associations between positive social interactions and emotion experiences and physical health outcomes in older adulthood. Our study will systematically test a biobehavioral model of social relationships and health, and examine a key peptide that may underlie the observed beneficial effects of positive social interactions and psychological states on health and longevity. At conclusion of the study we will have solid evidence concerning the effects of oxytocin on stress response, whether it has similar effects regardless of age or gender, and whether its potency depends in part on circulating levels of estradiol. Further work can build on this knowledge and consider potential effects of oxytocin on other biological parameters such as levels of inflammation or catecholamines.

Future research should examine how effects of oxytocin may relate to effects of different types of social support (friend, same sex, stranger, etc), and whether oxytocin may actually promote provision of more support or greater receptiveness to all kinds of support. A greater understanding of how oxytocin functions in human health may also be obtained by identification of gene by environment interactions in relation to oxytocin, and by considering the relation between oxytocin, the opioid pathway and endogenous opioids.

This novel area of investigation has the potential to have major impact on understanding how positive social and emotional experiences influence adult stress response, health, and longevity. This research will provide a solid platform from which to launch a larger interdisciplinary research program aimed at identifying neurobiological underpinnings for the observed epidemiological associations between positive social interactions and emotion experiences and physical health outcomes in older adulthood. A neurobiological understanding of resilience can inform efforts for both prevention and intervention of diseases or problems common in later life.

## Competing interests

The authors declare that they have no competing interests.

## Authors' contributions

All authors contributed to the design and coordination of the study and have read, commented on, and approved the manuscript.

## Pre-publication history

The pre-publication history for this paper can be accessed here:

http://www.biomedcentral.com/1471-2458/9/481/prepub
